# Cerebral complexity preceded enlarged brain size and reduced olfactory bulbs in Old World monkeys

**DOI:** 10.1038/ncomms8580

**Published:** 2015-07-03

**Authors:** Lauren A. Gonzales, Brenda R. Benefit, Monte L. McCrossin, Fred Spoor

**Affiliations:** 1 Department of Evolutionary Anthropology, Duke University, 104 Biological Sciences Building, Box 90383, Durham, North Carolina 27708-9976, USA; 2 Department of Anthropology, New Mexico State University, PO Box 30001, Las Cruces, New Mexico 88003-8001, USA; 3 Department of Human Evolution, Max Planck Institute for Evolutionary Anthropology, Leipzig 04103, Germany; 4 Department of Cell and Developmental Biology, UCL, Gower Street, London WC1E 6BT, UK

## Abstract

Analysis of the only complete early cercopithecoid (Old World monkey) endocast currently known, that of 15-million-year (Myr)-old *Victoriapithecus*, reveals an unexpectedly small endocranial volume (ECV) relative to body size and a large olfactory bulb volume relative to ECV, similar to extant lemurs and Oligocene anthropoids. However, the *Victoriapithecus* brain has principal and arcuate sulci of the frontal lobe not seen in the stem catarrhine *Aegyptopithecus*, as well as a distinctive cercopithecoid pattern of gyrification, indicating that cerebral complexity preceded encephalization in cercopithecoids. Since larger ECVs, expanded frontal lobes, and reduced olfactory bulbs are already present in the 17- to 18-Myr-old ape *Proconsul* these features evolved independently in hominoids (apes) and cercopithecoids and much earlier in the former. Moreover, the order of encephalization and brain reorganization was apparently different in hominoids and cercopithecoids, showing that brain size and cerebral organization evolve independently.

## Abstract (web summary)

The evolution of the brain in Old World monkeys (cercopithecoids) is poorly understood. Here the authors describe a complete endocast of *Victoriapithecus*, a 15 Myr old cercopithecoid, which shows that the brain size was much smaller and the olfactory bulbs much larger than in any extant catarrhine primate.

## Graphical abstract

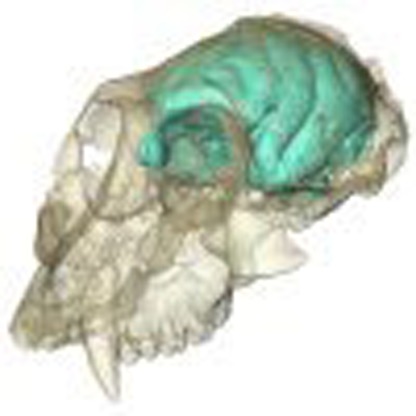

The relationship between the external morphology, cytoarchitecture and function of the brain is better understood for macaques than for other non-human primates because of their extensive use in neuroscience research^1^,^2^,^3^,^4^. However, it is not known when and in what order cercopithecoids evolved their distinctive pattern of cerebral sulci, brain size, relative size of major brain structures such as the olfactory bulbs, and inferred sensory and behavioural adaptations. Until now, the absence of complete hominoid and cercopithecoid cranial fossils from between 32 and 7 Myr ago necessitated a reliance on phylogenetic comparative studies of living primates, fossils outside this time period or incomplete fossils to reconstruct such events^5^,^6^. Such evidence indicated that the last common ancestor of cercopithecoids and hominoids had a small olfactory bulb and enhanced visual system, reflecting a change from reliance on olfactory to visual reproductive signalling^7^,^8^,^9^. In the absence of fossil evidence, disagreements persist as to whether increased brain size precedes, follows or evolves independently from increased gyrification and brain reorganization^10^,^11^,^12^,^13^,^14^.

The well-preserved 15-Myr-old adult male cranium KNM-MB 29100 of *Victoriapithecus* from Maboko Island, Kenya currently includes the only intact neurocranium of a Miocene catarrhine before 6 Myr^15^. *Victoriapithecus* represents a cercopithecoid clade that postdates the earliest fossil hominoid (*Rukwapithecus*) and cercopithecoid (*Nsungwepithecus*) by 10 million years (Myr); however, retention of a crista obliqua on the upper molars indicates that it is more primitive than the last common ancestor of extant Colobinae and Cercopithecinae^16^,^17^. Using high-resolution computed tomography (CT) we digitally extracted and reconstructed the endocast of KNM-MB 29100 to assess its bearing on the evolutionary relationship between brain size and complexity in the cercopithecoid lineage, and catarrhines in general.

## Results

### Endocranial volume

**Figure 1:**
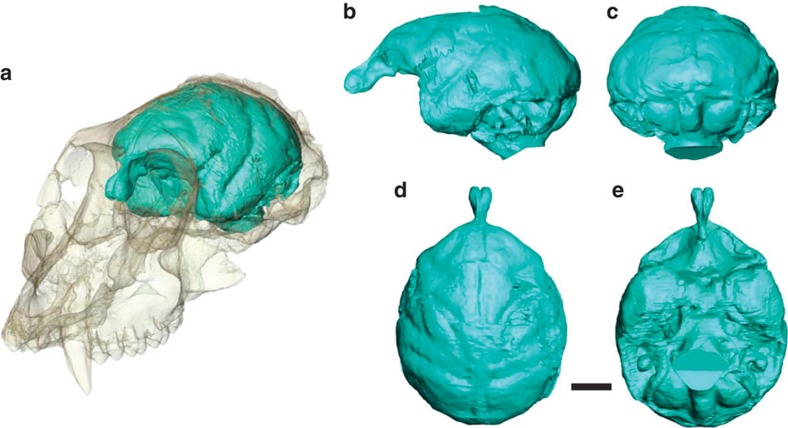
Endocast of *V. macinessi* (KNM-MB 29100). (**a**) Three-quarter view, shown inside the cranium rendered transparent; (**b**) lateral; (**c**) posterior; (**d**) superior and (**e**) inferior (basal) views. Scale bar, 1 cm.

**Figure 2:**
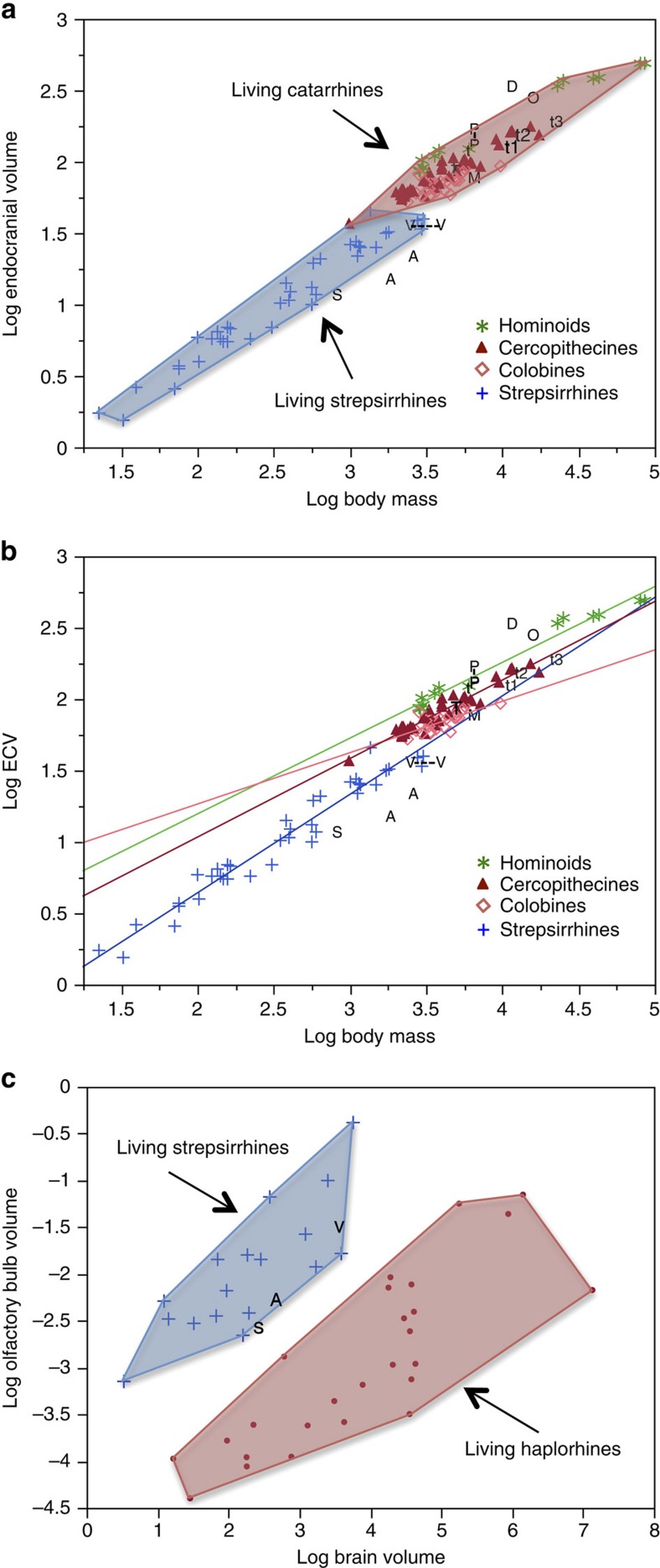
Analysis of the brain and olfactory bulb size. (**a**) Bivariate double logarthmic plot of ECV in cm^3^ against body mass in g for extant strepsirrhines and catarrhines from Isler^21^, with data superimposed for *Victoriapithecus* (V), Oligocene *Simonsius* (S)^22^ and *Aegyptopithecus* (A)^23^; Miocene hominoids *Proconsul* (P)^24^,^25^,^26^,^27^, *Turkanapithecus* (T)^25^,^26^, *Dryopithecus* (D)^25^,^26^,^28^, *Oreopithecus* (O)^25^,^26^,^29^,^30^,^31^,^32^,^33^; the Miocene colobine *Mesopithecus* (M)^5^,^18^; Plio-Pleistocene cercopithecines *T. darti* (t1)^18^,^35^, *T. brumpti* (t3)^35^,^36^; and *T. oswaldi* (t2)^18^,^35^,^37^,^38^; and extant *T. gelada*^18^,^35^. Maximum convex polygons are fit to species means. (**b**) Ordinary least squares regressions for strepsirrhines, colobines, cercopithecines and hominoids using the data in **a**. (**c**) Double logarithmic plot of olfactory bulb volume against brain volume for extant primates^43^,^45^ and *Victoriapithecus*. Brain volume data for V, S^22^ and A^23^ are represented by ECV in cm^3^. Olfactory bulb volume for fossil specimens is represented by olfactory fossa volume in cm^3^ with maximum convex polygons fit to species means.

The endocast of KNM-MB 29100 is well preserved and shows remarkably clear impressions of the cerebral sulci and gyri (Fig. 1; Supplementary Movie 1). After correcting for some distortions, an endocranial volume (ECV) of 35.6 cm^3^ was obtained (Supplementary Fig. 1), substantially less than the 54 cm^3^ previously inferred^15^. Body mass estimates on the basis of the cranial dimensions of KNM-MB 29100 converge between 6 and 7 kg (ref. 18), although its upper molar dimensions are among the largest sampled for the species and indicate the individual was closer to 10.5 kg (ref. 18). In comparison, the largest postcranial estimates of body mass for *Victoriapithecus* do not extend higher than 5.0–5.5 kg (refs 19, 20). In this study we use a conservative body mass range of 5–7 kg for KNM-MB 29100 as a compromise between these estimates. Relative to this body mass range, the newly measured *Victoriapithecus* ECV places the large male below the range of all known extant and fossil crown catarrhines (Supplementary Table 1, Fig. 2a)^21^,^22^,^23^,^24^,^25^,^26^,^27^,^28^,^29^,^30^,^31^,^32^,^33^,^34^,^35^,^36^,^37^,^38^. *Victoriapithecus* falls just below the best-fit regression line for extant strepsirrhines when assuming a postcrania-based body mass of 5 kg (Fig. 2b), and further below that line (overlapping *Indri*) when using a body mass of 6–7 kg for KNM-MB 29100 (ref. 21). ECVs for the Oligocene stem catarrhine *Aegyptopithecus* and stem anthropoid *Simonsius* (=*Parapithecus*) fall somewhat further below the strepsirrhine regression than *Victoriapithecus,* indicating that 15 Myr ago the latter had only a slightly larger relative brain size than 32-Myr-old Oligocene stem catarrhines^22^,^23^.

Among cercopithecoids *Miopithecus talapoin* has an ECV similar to *Victoriapithecus*, but an average body mass of 1.5–1.9 kg (ref. 21). Cercopithecoids with slightly lower body masses than predicted for KNM-MB 29100, between 4 and 5 kg, have ECVs roughly twice that of *Victoriapithecus* ranging from 51 to 82 cm^3^ (average 65.6, *n*=6 species) if female and 53–71 cm^3^ (average 63, *n*=11 species) if male^21^. Of these species, colobine monkeys have the smallest ECVs relative to body mass, a phenomenon related to their folivorous diet^21^,^37^. Since dental morphology and microwear indicate that *Victoriapithecus* was clearly frugivorous, diet did not contribute to its extremely small brain size^16^.

Compared with *Victoriapithecus*, the estimated ECV for the 8- to 9-Myr-old colobine *Mesopithecus* is substantially larger, falling within the extant catarrhine cluster but just below the modern colobine regression line relative to its inferred body mass^5^,^18^ (Fig. 2a,b). Directly measured ECVs for four species of the Plio-Pleistocene cercopithecine *Theropithecus* are similarly larger relative to body mass than *Victoriapithecus* but are all smaller than living *T. gelada,* and plot towards the edge of the catarrhine cluster where species with the smallest ECVs per body mass are found^35^,^37^(Fig. 2a). The limited fossil cercopithecoid data indicate that ECV had increased in late-Miocene and Plio-Pleistocene Old World monkeys relative to the very small volume found in middle Miocene *Victoriapithecus.* While ECV increases evolved independently in colobine and cercopithecine subfamilies, neither lineage had reached modern ECV level until the Holocene.

Existing evidence indicates that Miocene apes (excluding *Afropithecus*) were substantially more encephalized than contemporary cercopithecoids, although assessing exactly how different their ECVs were will require an improved fossil record. ECV estimates for incomplete skulls of 17- to 18-Myr-old *Proconsul*^24^,^25^,^26^,^27^,10-Myr-old *Dryopithecus*^25^,^26^,^28^ and 8-Myr-old *Oreopithecus*^25^,^26^,^29^,^30^,^31^,^32^,^33^, obtained from regressions of extant anthropoid cranial dimensions against ECV, all fall either within the range of great apes, hylobatids or cercopithecoids (lower end) when considered relative to estimated body mass^5^,^22^,^23^,^24^ (Fig. 2a). However, since similar methods^15^ overestimated the ECV of *Victoriapithecus* by 34% compared with the direct measurements obtained here, these apparently large hominoid ECV estimates could be an artefact of methodology. The earliest conclusive evidence that hominoids reached ECV levels of extant apes comes from the late-Miocene *Sahelanthropus* cranium, for which ECV falls within the range of chimpanzees relative to its estimated body mass^39^,^40^,^41^.

### Olfaction

*Victoriapithecus* differs from modern anthropoids in having much larger olfactory bulbs that project anteriorly as in strepsirrhines and *Aegyptopithecus*^23^,^42^ (Supplementary Fig. 2). Relative to ECV, the olfactory fossa volume is large (0.22 cm^3^), falling within the lower range of strepsirrhines and upper-most range of anthropoids, similar to *Aegyptopithecus* and *Simonsius* (Fig. 2c)^22^,^23^,^43^. Therefore, olfactory bulb reduction must have occurred in cercopithecoids after 15 Myr, although it is already reduced in the 17- to 18-Myr-old hominoid *Proconsul*^44^. Measurement of a large olfactory bulb relative to the brain size in *Aegyptopithecus* had previously demonstrated that olfactory bulb reduction occurred independently in platyrrhine and catarrhine primates^23^,^43^,^45^,^46^. The *Victoriapithecus* evidence further reveals that olfactory reduction was not present in the last common ancestor of hominoids and cercopithecoids, but instead evolved independently in these two clades and at least 2 Myr later in cercopithecoids than hominoids.

**Figure 3:**
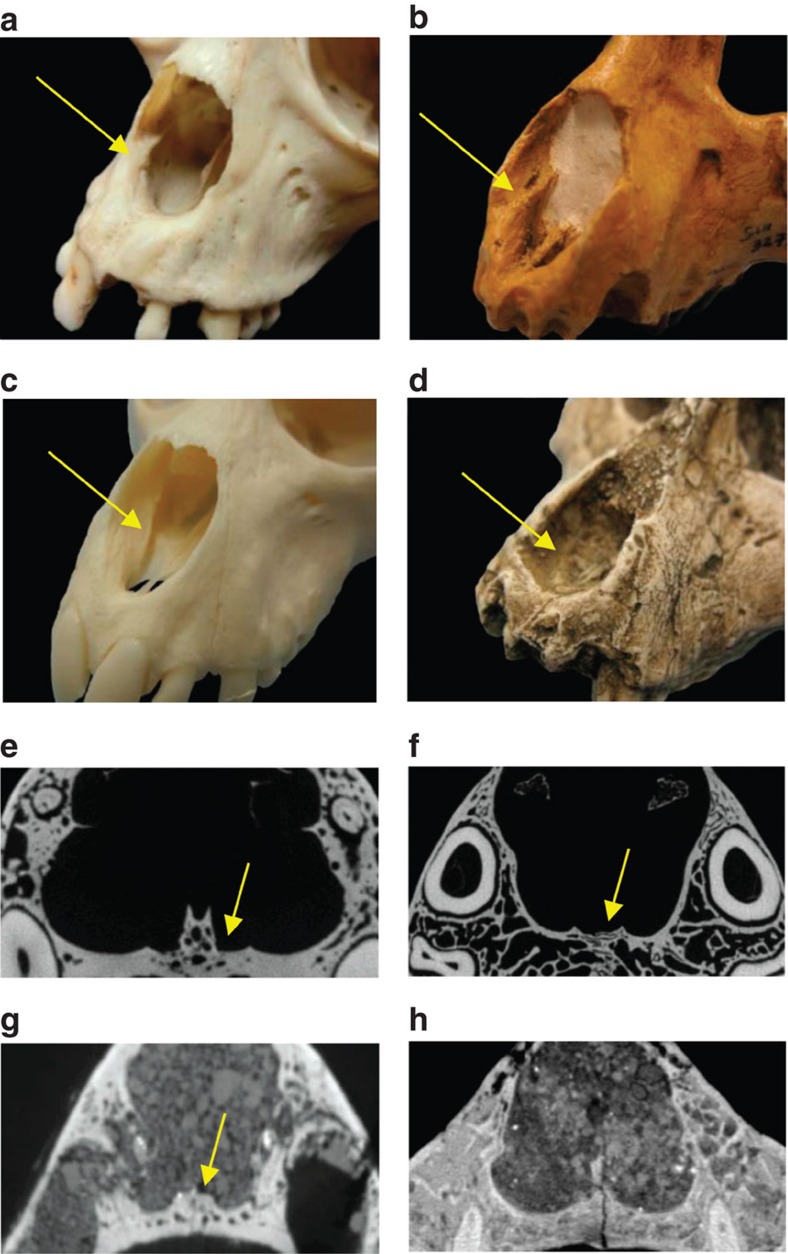
Interior nasal anatomy and vomeronasal complex in primates. Atrioturbinal ridges extend outwards on the premaxilla in (**a**) *Ateles* and (**b**) *Aegpyptopithecus* (arrows) but are absent in (**c**) *Macaca* and (**d**) *Victoriapithecus*, whose nasal ridges descend inferiorly and terminate within the nasal cavity. The vomeronasal groove (VNG) is a U- or J-shaped depression (arrows) along the bony maxillary in palate^47^ in (**e**) *Potto*, (**f**) *Alouatta* and (**g**) the stem catarrhine *Aegyptopithecus* but is absent in (**h**) *Victoriapithecus*.

Although the olfactory bulbs of *Victoriapithecus* are larger compared with those of crown catarrhines, and more similar in size to *Aegyptopithecus*, its olfactory system may have differed significantly from the latter. Mammalian olfaction consists of two distinct parts, the main olfactory bulb, which is typically used to detect volatile odorant molecules, and the vomeronasal organ (VNO) used to detect odorant molecules of high molecular weight such as water-soluble pheromones^47^. Two bony structures associated with a functioning VNO in extant strepsirrhines, tarsiers and platyrrhines, an atrioturbinal ridge in the nasal complex and a vomeronasal groove along the maxillary palate, are present in *Aegyptopithecus* but absent in *Victoriapithecus* and living crown catarrhines^47^,^48^,^49^,^50^,^51^ (Fig. 3a–d). Miocene hominoids *Afropithecus* and *Proconsul* similarly lack VNO-related structures; however, the presence of an atrioturbinal ridge in two small-bodied early Miocene non-cercopithecoid catarrhines *Limnopithecus* and *Kalepithecus* indicates that some catarrhine lineages retained VNO function during the Miocene^48^. In contrast to *Aegyptopithecus* and these small-bodied catarrhines *Victoriapithecus, Afropithecus* and *Proconsul* would have relied only on their main olfactory bulbs rather than VNO for the detection of socially relevant olfactory stimuli, possibly including pheromones (Fig. 3e–h)^52^,^53^,^54^,^55^.

### Cerebral organization

**Figure 4:**
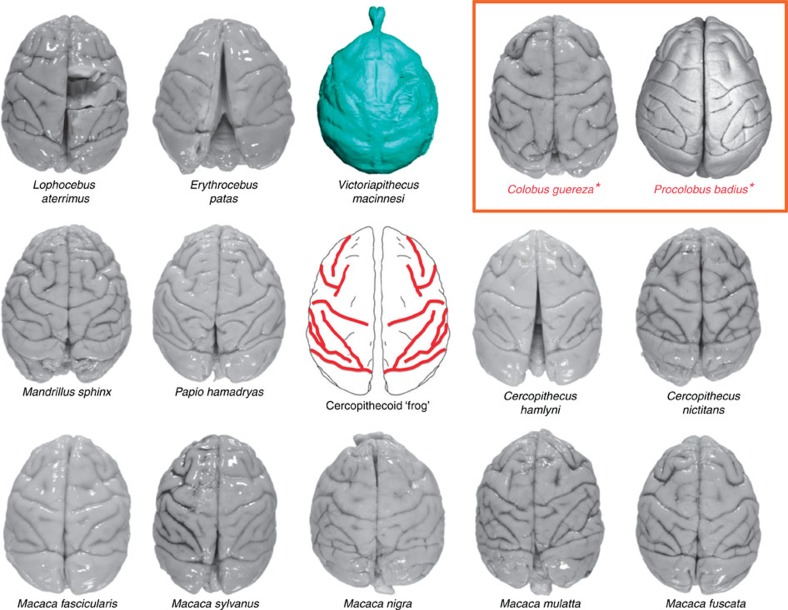
Superior views of extant cercopithecoid brains compared with *Victoriapithecus* endocast. All display the distinctive pattern we describe as ‘frog-like' (centre image). Orange box marks the colobine species. Brains not scaled to actual size. Brain images from the Primate Brain Bank, Netherlands Institute for Neuroscience, the Netherlands, except *Procolobus badius* which was provided by K. Zilles.

**Figure 5:**
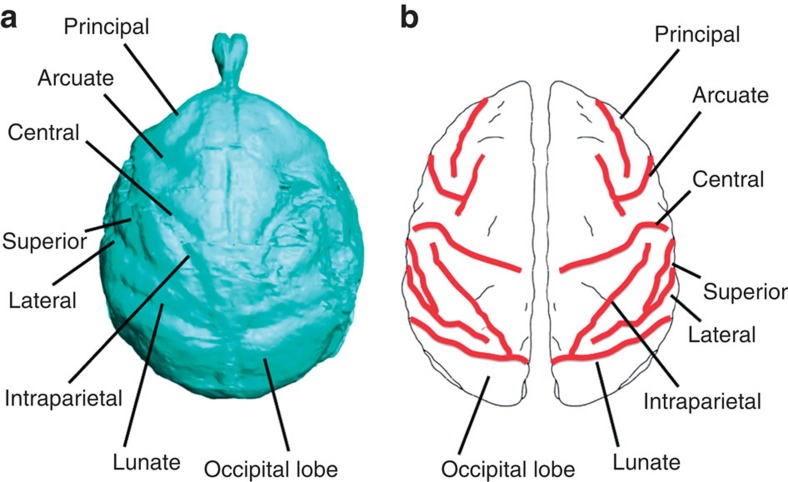
Sulci on the superior aspect of the *Victoriapithecus* endocast. (**a**) CT-based reconstruction and (**b**) line drawing highlighting the basic sulcal configuration representative of all cercopithecoids.

**Figure 6:**
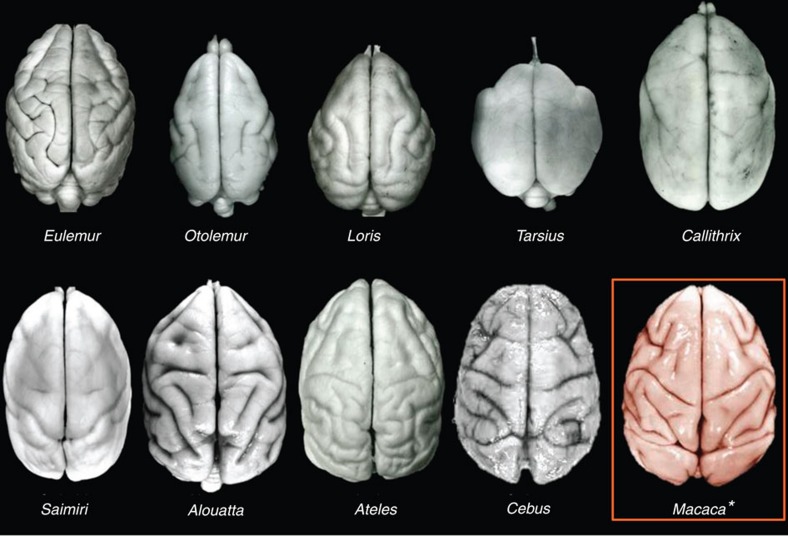
Comparison of sulcal patterns in primates. Superior views of strepsirrhines (*Eulemur, Otolemur* and *Loris*), *Tarsius*, and platyrrhines (*Callithrix, Saimiri, Alouatta, Ateles* and *Cebus*) compared with the cercopithecoid *Macaca*. Among extant primates, only *Cebus* converges on the cercopithecoid sulcal pattern. Brain images provided by K. Zilles except for *Cebus* and *Macaca* which are from the Primate Brain Bank, Netherlands Institute for Neuroscience, the Netherlands.

Notwithstanding its small ECV and large olfactory bulbs, the cerebral cortex of *Victoriapithecus* is reorganized relative to Oligocene anthropoids and exhibits the modern cercopithecoid pattern of sulci and gyri. In superior view, the sulci of all cercopithecoids, including *Victoriapithecus,* are arranged in a highly distinctive frog-shaped pattern (Fig. 4). The arms of the frog are formed by the principal and arcuate sulci that demarcate the prefrontal cortex; the central sulcus (primarily an anthropoid trait) borders the frontal cortex posteriorly forming the top of the frog's thigh; the intraparietal sulcus separates the back of the frog's thigh from the calf; the superior temporal sulcus forms the frog's shin; and the lunate sulcus forms the bottom of its foot and borders the occipital lobe anteriorly (Fig. 5). No other primate has this cercopithecoid sulcal pattern with the exception of the platyrrhine *Cebus* in which it convergently evolved^5^,^6^,^56^,^57^ (Fig. 6). Unlike variation seen in platyrrhine sulcal patterns, the frog-shaped pattern is highly conserved across cercopithecoids, although some differences exist between the two subfamilies^56^,^57^.

In contrast to *Victoriapithecus, Aegyptopithecus* has smooth and featureless frontal lobes lacking both principal (=rectus) and arcuate sulci^5^,^23^,^42^ as in strepsirrhines and platyrrhines excluding *Cebus. Aegyptopithecus* also has smooth occipital and temporal lobes lacking inferior occipital sulci, dimpling of the temporal lobes and anterior and posterior middle temporal sulci, all of which occur in *Victoriapithecus*. Principal sulci are shared by all crown catarrhines and must have been retained from an ancestral condition more recent than *Aegyptopithecus*. The presence of arcuate sulci in *Victoriapithecus*, all other cercopithecoids, the 10-Myr-old hominoid *Dryopithecus*^25^, extant great apes and humans indicates they may have been present in the eucatarrhine common ancestor. The unique and complex pattern of sulci occurring in the prefrontal region of hylobatids and the early Miocene *Proconsul*^5^,^58^ could therefore be interpreted as derived, although their lack of an arcuate sulcus has previously been interpreted as primitive^5^,^25^,^56^,^58^. Alternatively, the arcuate sulcus might have evolved independently and convergently in hominoids and cercopithecoids, as it did in *Cebus*. In macaques, areas around the arcuate sulcus are involved in visual working memory, hand–eye coordination and mirror neurons activated by observing the movements of others^59^. Neurons in the macaque inferior temporal lobe are involved in visual pattern recognition including the processing of colours as well as place, face and object recognition^59^,^60^. In particular, the posterior middle temporal sulci, found in *Victoriapithecus* but not in *Aegyptopithecus*, concerns an area where sharply tuned colour-selective neurons are concentrated in macaque brains^60^. Therefore, the presence of additional temporal lobe sulci in *Victoriapithecus* compared with Oligocene anthropoids suggests that it had already evolved a more complex visual system than *Aegyptopithecus* in spite of their similarly small ECVs.

Differences between *Victoriapithecus* and extant cercopithecoid cerebral cortices are indicated by the more anterior position of various sulcal landmarks relative to endocast length and height in the former (Supplementary Table 2). *Victoriapithecus* shares with *Aegyptopithecus* the lack of an obvious precentral superior sulcus, and frontal lobes that are more V-shaped anteriorly, shorter anteroposteriorly and lower relative to length than in extant cercopithecoids. Among the Miocene apes, the frontal lobes of *Proconsul* (17–18 Myr)*, Turkanapithecus* (17 Myr) and *Dryopithecus* (10 Myr) are substantially broader and less constricted anteriorly than both *Aegyptopithecus* and *Victoriapithecus*. Only *Afropithecus* (17 Myr) has a small V-shaped frontal cortex among Miocene hominoids.

The *Victoriapithecus* motor cortex (the frog's thigh) appears to have been narrower and the superior temporal gyrus shorter than in extant cercopithecoids as indicated by the more anterior position of the confluence of lateral and superior temporal sulci. In addition, the inferior temporal lobe is large and uniquely has a large posterior inferior temporal region that is continuous with the occipital lobe, creating a distinct thickening in the occipital lobe between the short lunate and upwardly curving inferior occipital sulcus that is much less anteriorly positioned than in any extant cercopithecoid we observed.

**Figure 7:**
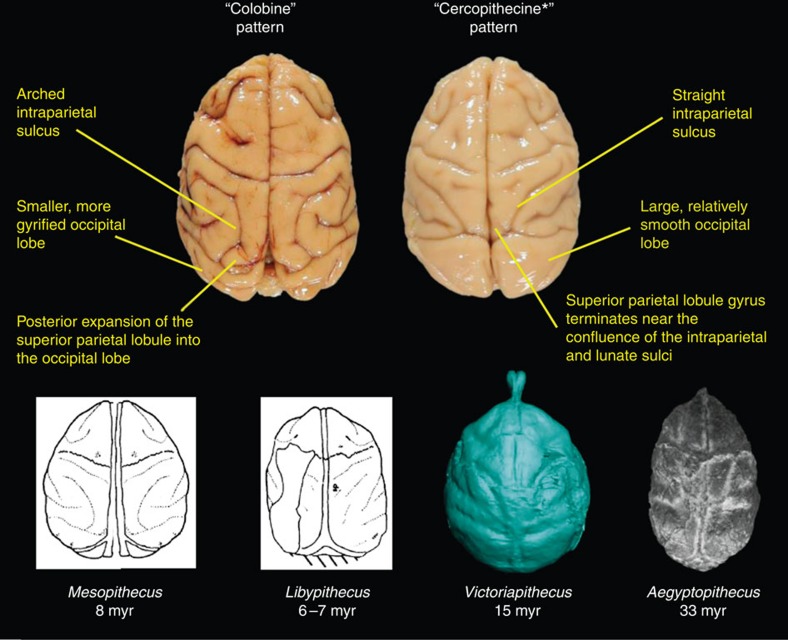
Differences between extant colobine and cercopithecine superior parietal lobes. Superior views of the brains of *Colobus guereza* (left) and *Macaca fascicularis* (right) show that owing to posterior expansion of the SPL in colobines, the intraparietal sulcal appears to be arched in lateral view, whereas in the cercopithecine the intraparietal sulcus is straight. *Aegyptopithecus*^42^ and the fossil colobine *Libypithecus*^5^ appear to have straight intraparietal sulci, whereas *Mesopithecus*^5^ has an arched intraparietal sulcas and some expansion of SPL. Both *Mesopithecus* and *Libypithecus* have anteroposteriorly short occipital lobes, unlike *Victoriapithecus* and *Aegyptopithecus*. Extant cercopithecoid brain images were provided by the Primate Brain Bank, Netherlands Institute for Neuroscience, the Netherlands.

Of the two extant cercopithecoid subfamilies, the *Victoriapithecus* cerebral cortex is more similar to those of cercopithecines than colobines. Only extant colobines and the late-Miocene colobine *Mesopithecus* have intraparietal sulci that diverge laterally at their posterior ends as the superior parietal lobule (SPL) expands and impinges on a shorter but more gyrified occipital lobe^56^,^57^. *Victoriapithecus* shares with extant cercopithecines and the late-Miocene colobine *Libypithecus* intraparietal sulci that are straight and converge posteriorly as they approach the lunate sulcus, posterior ends of the superior parietal gyrus that are V-shaped and a more anteriorly positioned lunate sulcus^5^ (Fig. 7). Because *Aegyptopithecus* and *Victoriapithecus* share relatively large occipital lobes and unexpanded SPLs with cercopithecines (Supplementary Table 2), it is likely that the colobine condition is derived^56^. Lateral and superior temporal sulci converge in *Victoriapithecus* as in most cercopithecines, and some colobines including *Libypithecus* and *Semnopithecus,* but remain separate in most colobines, and some fossil cercopithecines including *Paradolichopithecus arvernensis*^5^ and *T. oswaldi*^56^,^57^,^61^. The only trait *Victoriapithecus* shares uniquely with colobines is asymmetry of the prefrontal cortex resulting from a superiorly directed extension from the principal sulcus occurring only on the right side in the Miocene monkey^56^.

## Discussion

**Figure 8:**
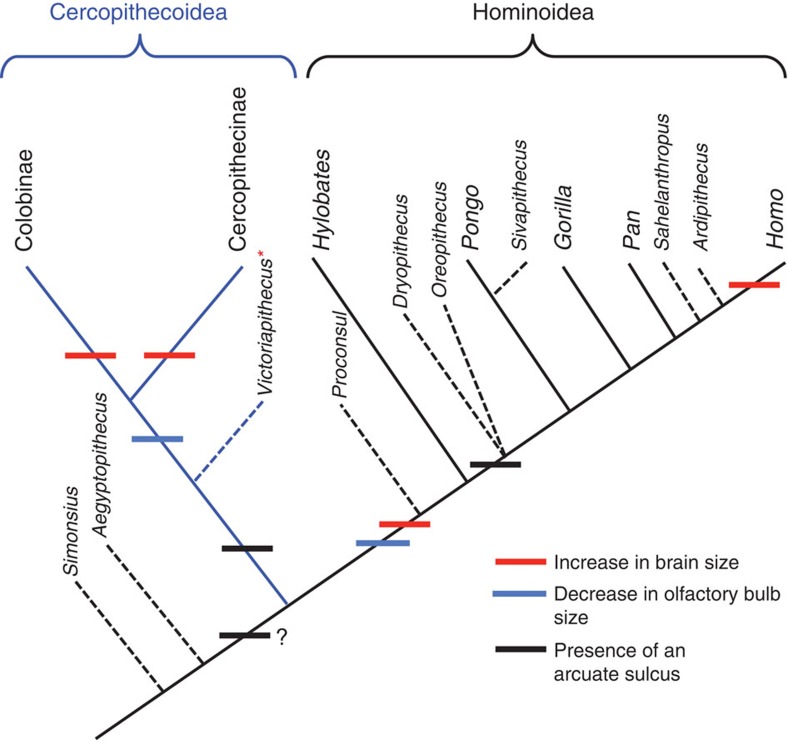
Cladogram of fossil and extant taxa catarrhines discussed in this paper. The appearance of major brain changes discussed in this paper are indicated.

The combination in *Victoriapithecus* of modern cercopithecoid cerebral complexity and gyrification with a strepsirrhine-like small ECV and large olfactory bulbs is unexpected in an Old World monkey that postdates the hominoid/cercopithecoid divergence by 10–15 Myr (Fig. 8). This is especially true because encephalization has been linked with increased gyrification in anthropoid evolution, and in particular in the genus *Homo*^10^,^11^,^62^. However, recent evidence that brain size and gyrification are controlled by different genes in catarrhine primates suggests that encephalization and cerebral complexity could evolve independently^14^, and that either one could precede the other. The discovery that complex gyrification evolved before increased brain size in cercopithecoids underlines the finding that the notably small but highly gyrified brain of the tool-making hominin *H. floresiensis*^63^ is perhaps not as remarkable as it may seem. Diversity in the patterning of encephalization and gyrification is also seen in the evolutionary history of terrestrial and aquatic cetartiodactyls with encephalization preceding gyrification in cetaceans, but gyrification preceding encephalization in terrestrial artiodactyls^64^,^65^.

Following the evolution of the distinctive frog-like pattern of cercopithecoid sulci in *Victoriapithecus* by the middle Miocene, it was retained in both colobine and cercopithecine subfamilies resulting in far less intergeneric sulcal variation in extant Old World monkeys than is seen in hominoids, platyrrhines and strepsirrhines^56^. We are uncertain why this pattern was so successful that it remained static for the past 15 Myr, but it convergently evolved in *Cebus* monkeys that are among the most intelligent of platyrrhines^66^. Sulcal differences between colobines and cercopithecines are restricted to greater asymmetry of prefrontal sulci and SPL expansion with related changes in the intraparietal sulcus in colobines. We hypothesize that SPL expansion in colobines may be an adaptation for folivory since in macaques V6 and PE regions in that area appear to be devoted to proprioception and the reaching and grasping of objects such as occurs for prolonged periods during the harvesting of leaves^67^,^68^. Similar expansion of the SPL is seen in extant hylobatids, which are known to include large amounts of leaves in their diets^55^,^69^, but does not occur in highly frugivorous *Aegyptopithecus, Victoriapithecus* or extant cercopithecine monkeys^42^,^56^.

Convergent evolution appears to have been a hallmark of catarrhine brain evolution, with reduction in olfactory bulb volume, widening and expansion of the frontal lobe and increased ECV having evolved independently in hominoids and cercopithecoids as well as in colobines and cercopithecines. In addition, the absence of the arcuate sulcus in *Proconsul* and hylobatids indicates that this sulcus may not have been present in the last common ancestor of hominoids and cercopithecoids, in which case it convergently evolved in cercopithecoids and hominids. Fossil evidence has already shown that the prefrontal cruciate sulcus evolved independently in five major carnivore clades, indicating that convergent evolution of prefrontal sulci such as the arcuate sulcus is possible^70^. Alternatively, absence of the sulcus in *Proconsul* and hylobatids may represent a convergent loss or a shared derived condition.

The timing of olfactory bulb reduction and increased ECV appears to have been very different in cercopithecoids than hominoids. Existing evidence indicates that reduction of the olfactory bulb and evolution of modern catarrhine ECV levels had evolved by 17–18 Myr ago in *Proconsul*^24^,^25^,^26^,^27^,^44^. In contrast, olfactory bulb size and ECV in 15-million-year-old cercopithecoids had changed only slightly relative to 32-million-year-old Oligocene anthropoids. Late-Miocene and Plio-Pleistocene monkeys approach modern cercopithecoid ECV levels; however, Old World monkey brains did not fully reach their extant size until the Holocene^5^,^21^,^35^,^37^, with increased ECV evolving independently in colobines and cercopithecines. A pattern of ECV increase similar to that of cercopithecoids occurred in terrestrial artiodactyls, for which ECV relative to body size changed little between the Oligocene and Miocene, but increased dramatically during the Holocene and did so independently in several different lineages^65^.

In conclusion, differences in the brain and cognitive evolution between hominoids and cercopithecoids can now be traced back to the early Miocene (17–18 Myr). With cercopithecoids showing cerebral and visual system complexity preceding encephalization and olfactory bulb reduction, and hominoids exhibiting frontal lobe expansion and encephalization before gyrification, it appears that some morphological and functional similarities between extant macaque and hominoid brains may have evolved convergently. The combination of a modern cercopithecoid sulcal pattern with strepsirrhine ECV and olfactory bulb size in *Victoriapithecus* refutes hypotheses that increased brain size is the major factor causing the development of cerebral complexity in anthropoids. Instead, evidence from the brain of *Victoriapithecus* shows that cerebral complexity and brain size, and changes in visual and olfactory systems, are influenced by different sets of selective pressures and therefore evolve independently.

## Methods

### CT scan information and visualization

KNM-MB 29100 was CT scanned with the BIR ACTIS 225/300 of the Max Planck Institute for Evolutionary Anthropology, Leipzig, at the time installed at the National Museums of Kenya in Nairobi. The isotropic voxel size is 0.044 mm. Avizo 7.1 and 8.0 (Visualization Sciences Group) and Geomagic Studio 2013 (Geomagic Inc.) were used for visualization, segmentation, reconstruction and quantification.

### Correction for cranial distortion

To calculate the ECV of KNM-MB 29100, the preserved endocast was corrected for distortion in three areas (Supplementary Fig. 1). The endocranial surface associated with the inferiorly depressed frontal squama was realigned with that of the parietals, filling smaller areas bilaterally by surface interpolation. The central part of both orbital roofs is fragmented and pushed superiorly. The associated surfaces were removed from the endocast and filled by interpolation based on the surrounding, well-preserved areas. Lastly, a small distorted area of the endocast associated with the left temporal lobe was interpolated. The ECV of the reconstructed endocast is 35.6 cm^3^. Since parts of the right frontoparietal area are not as well preserved as the left side, we also calculated ECV values for two endocast reconstructions based on the left half combined with its mirror image. One version uses an overall best-fit midsagittal plane, whereas the other applies the additional constraint that the original foramen magnum size is maintained. The associated ECVs, 35.5 and 36.2 cm^3^, respectively, bracket the value obtained for the full endocast.

## Additional information

**How to cite this article:** Gonzales, L.A. *et al.* Cerebral complexity preceded enlarged brain size and reduced olfactory bulbs in Old World monkeys. *Nat. Commun.* 6:7580 doi: 10.1038/ncomms8580 (2015).

## Supplementary Material

Supplementary Figures, Tables and References Supplementary Figures 1-2, Supplementary Tables 1-2 and Supplementary References

Supplementary Movie 1 The KNM-MB 29100 endocast rotating around a supero-inferior axis. The surface reconstruction, based on high-resolution CT images, is of the endocast as preserved in the fossil, without corrections for distortion.
