# Supplementary material for: Cerebral complexity preceded enlarged brain size and reduced olfactory bulbs in Old World monkeys
Source: Nat Commun. 2015 Jul 3;6:7580. doi: 10.1038/ncomms8580 (PMC4506532; doi:10.1038/ncomms8580)
Supplement: Supplementary Figures, Tables and References — Supplementary Figures 1-2, Supplementary Tables 1-2 and Supplementary References [file ncomms8580-s1.pdf]

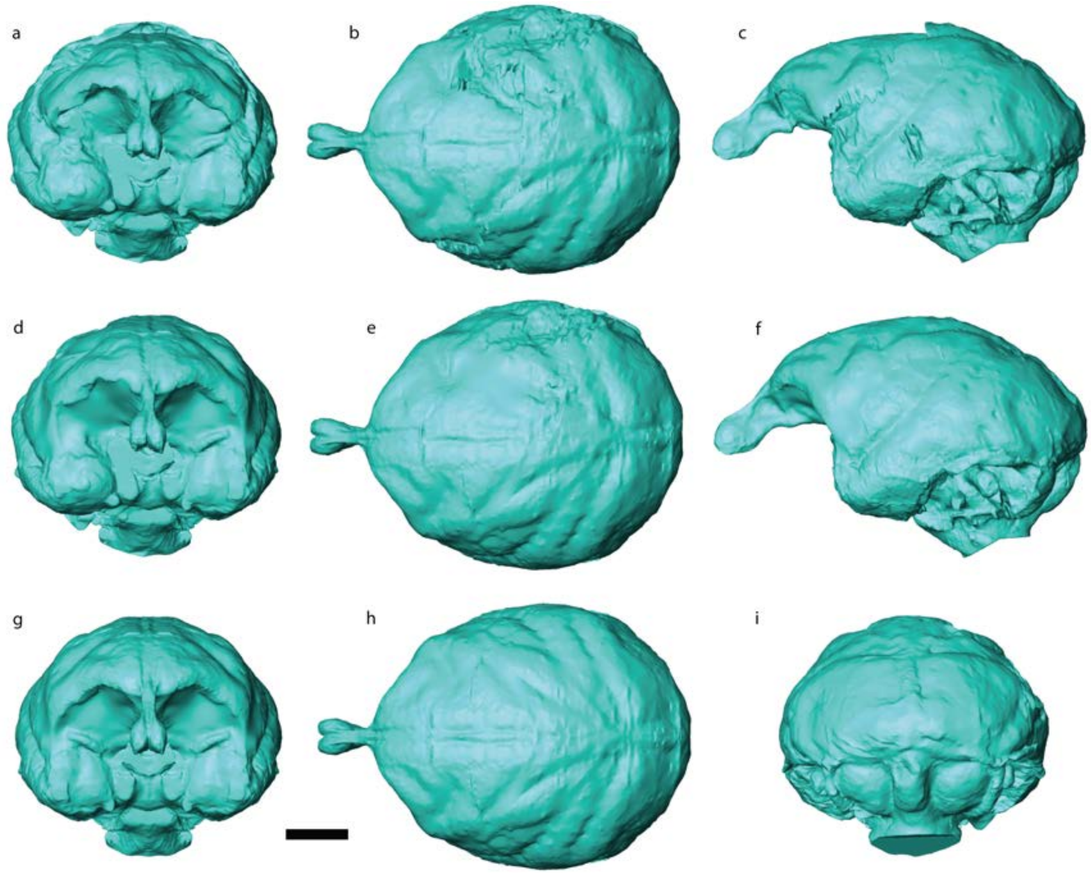

Supplementary Figure 1. Reconstruction of endocast of KNM-MB 29100.
a-c, Anterior, superior and left lateral views of the endocast as extracted from the CT images. **d-f**, Same views of the reconstructed endocast after repositioning the frontal squama and correcting the orbital roofs and smaller defects. **g-h**, Anterior and superior views after mirror-imaging the better preserved left half of the reconstructed endocast. **i**, posterior view of the endocast as extracted. Scale bar is 1 cm.

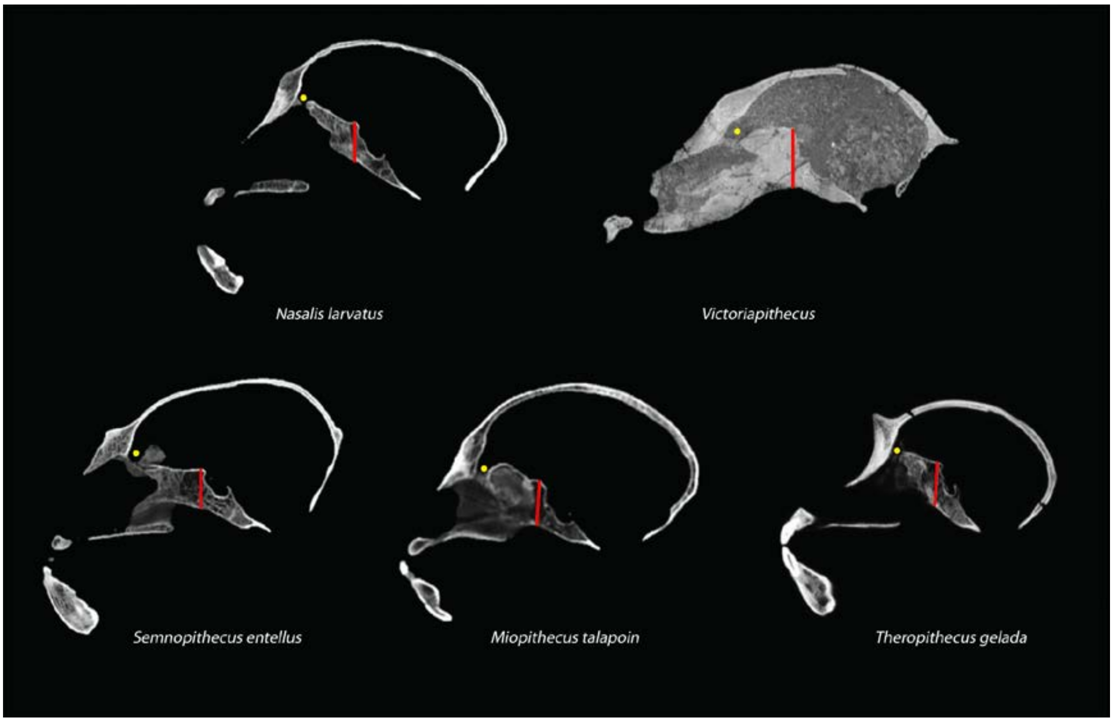

Supplementary Figure 2. The olfactory fossa of extant cercopithecoids and *Victoriapithecus.* Midsagittal CT images show that the fossa (yellow dot) is small and oriented inferiorly in extant species, but large and anteriorly oriented in *Victoriapithecus*. Note the lower height of the frontal in *Victoriapithecus*, as well as its inferosuperiorly tall sphenoidal body (red line).

Species	Specimen	Cranial Capacity in cm^3	Body Mass in kg	ER_{GA}[1]	EQ[2]
Aegyptopithecus zeuxis	CGM 40237 Male	20.5-21.8[3]	5.3[3]		
(~29-30 Myr) Fayum	CGM 85785 Female	14.56[3]	3.71[3]		
Proconsul heseloni	KNM-RU 7290	150[4]	10-11[1,2,5,6]	-0.46	1.94
(17 Myr) Rusinga	Female	**167.3**[4] (154-181)[5]	15 (**13**-18)[1,2]		
		130[1]			
Turkanapithecus kalakolensis (16-18 Myr) Kalodirr		84.3[1]	10[1]		
Dryopithecus brancoi	Rud 200 Female	330[2,7]	20.3-23.2[2]	0.01	2.35
(10 Myr) Rudabanya	Rud 77 Female	305[2,7]	28.7-31.2[2]		
Oreopithecus bambolii	IGF 11778 Male	**276**-579[8]	40[9]	-0.50	
(8 Myr) Baccinello		200[1,10]	**32**[11]		
	BAC 63 subadult	128 (83-173)[12]	22.5[12]		
			17.2[13]		
Sahelanthropus tchadensis	TM 266-01-060-1	320-380[14]	chimpanzee-sized[14]		
(6-7 Myr) Toros-Menalla	Male	360-**370**[15]	(~30-50)[16]		
		378[17]			
Ardipithecus ramidus	*ARA-VP-6/500*	300[18]	51[19]	-0.23	
(4.4 Myr) Aramis	*Female*				
Great Apes				0.00 (-0.05-0.09)	1.53-2.48
Lesser Apes				-0.73 (-.87-0.57)	1.93-2.74
Cercopithecoidea				-0.97 (-.37-0.46)	1.05-2.76
Mesopithecus pentelicus		70-75[4]	9-13[20]		
(8-9 Myr) Pikermi					
Theropithecus gelada	Male	130[21] (measured)	18.4 (16-20) [20]		
extant	Female	118[21] (measured)	11.9[20] (9-14)		
Theropithecus baringensis	BC 2, Male	129[21]	25[22]		
(3.2 Myr) Chemeron					
Theropithecus brumpti	WT 16828	187[21]	43[22]		
(3.1 Myr) Nachukui	Male		36[20]		
Theropithecus darti	MP 222 Female	122[21]	22[22]		
(3.0Myr) Makapansgat	M 3073 Female	143[20]	22[20]		
Theropithecus oswaldi	BM 32102	150[23] (measured)	20 (13-28)[20]		
(~2 Myr) Kanjera	BM 14836 Female	140[21],154[23] (measured)	26.4-29.8[24]		
Theropithecus oswaldi	SK 561 Female	150[21] (measured)	24-25[20]		
(1.6 Myr) Swartkrans					
Theropithecus oswaldi	KNM-ER 969 ?sex	145[21] (measured)	Female 26 (18-39)[20]		
(1.6 Myr) Koobi Fora			Male **36**-72[20]		
Theropithecus oswaldi	DAT 600/82	200[23] (measured)	32.5-38.7[24]		
(1.5 Myr) Peninj			30[20]		

Supplementary Table 1. Estimated ECV, Body Mass, Encephalization Residuals, and Encephalization Quotient for fossil catarrhines. With the exception of ECVs for *Aegyptopithecus, Sahelanthropus, Ardipithecus,* and most *Theropithecus* ECVs for incomplete or distorted crania of other catarrhines are estimated based on the chord length of the skull from nasion to inion (*Proconsul* and *Dryopithecus*), foramen magnum area (*Proconsul, Turkanapithecus, Oreopithecus*), other cranial dimensions and/or visual comparison with living catarrhines (*Proconsul, Oreopithecus, Mesopithecus*). Estimated body weights derive from cranial measurements including orbit area, the average of postcranial estimates for the same sex of that species, or associated postcrania thought to belong to the same individual. For species with multiple estimates, the ECV estimate we view as most reliable is given in bold.

Species	Frontal lobe L/L	Frontal lobe H/H	Confluence of LS and STS/L	Lunate Sulcus/l
Semnopithecus entellus	53.5	46.5	72.1	95.4
Macaca mulatta	57.5	41.5	67.5	82.5
Cercocebus torquatus	52.3	44.4	61.4	72.7
Mandrillus sphinx	57.7	45.8	60.2	90.1
Papio papio	60.9	44.2	71.7	91.3
Cercopithecus nictitans	55.6	35.7	62.2	82.2
Victoriapithecus macinnesi	48.0	35.7	52.0	70.0
Aegyptopithecus zeuxis	32.0	29.8		69.1

Supplementary Table 2. Position of cerebral cortex landmarks in fossil and extant cercopithecoids. Frontal lobes of *Victoriapithecus* are intermediate in size between *Aegyptopithecus* and extant cercopithecoids as indicated by frontal lobe length (anterior-most point of endocast to posterior-most point, usually superior, of the central sulcus) against endocast length, and frontal lobe height (maximum vertical height between the lateral sulcus inferiorly and central sulcus superiorly) against endocast height. The confluence of lateral and superior temporal sulcus is positioned more anteriorly in *Victoriapithecus* than in extant Old World monkeys relative to endocast length. *Victoriapithecus* and *Aegyptopithecus* have more anteriorly positioned lunate sulci than extant cercopithecoids, indicative of their larger occipital lobes. Measurements were taken from photographs of brains or endocasts in lateral view.

Supplementary References

1. Alba, D. M. Cognitive inferences in fossil apes (Primates, Hominoidea): does encephalization reflect intelligence? *J. Anthropol. Sci.* **88**, 11–48 (2010).

2. Begun, D. R. & Kordos, L. in *The Evolution of Thought. Evolutionary Origins of Great Ape Intelligence. (*eds. Russon, A. E. & Begun D. R.) 260-279 (Cambridge University Press, 2004).

3. Simons, E. L., Seiffert, E. R., Ryan, T. M., & Attia, Y. A remarkable female cranium of the early Oligocene anthropoid *Aegyptopithecus zeuxis*. *Proc. Natl. Acad. Sci. USA* **104**, 8731–8736 (2007).

4. Radinsky, L. The fossil evidence of anthropoid brain evolution. *Am. J. Phys. Anthropol*. **41**, 15-28 (1974).

5. Walker, A. C., Falk, D., Smith, R., & Pickford, M. The skull of *Proconsul africanus*: reconstruction and cranial capacity. *Nature* **305**, 525-527 (1983).

6. Manser, J, & Harrison, T. Estimates of cranial capacity and encephalization in *Proconsul* and *Turkanapithecus*. *Am. J. Phys. Anthropol*. **28**, 189 (1999).

7. Kordos, L. & Begun, D. R. A new cranium of *Dryopithecus* from Rudabanya, Hungary. *J. Hum. Evol*. **41**, 689-700 (2001).

8. Straus, W. L. & Schon, M. A. Cranial capacity of *Oreopithecus bambolii*. *Science* **132**, 670-672 (1960).

9. Schultz, A. H. Einige Beobachtungen und Masse am Skelett von *Oreopithecus* im Vergleich mit anderen catarrhinen Primaten. *Z. Morph. Anthropol*. **50**, 136 (1960).

10. Szalay, F. & Berzi, A. Cranial anatomy of *Oreopithecus*. *Science* **180**, 183-185 (1973).

11. Jungers, W.L. Body size and morphometric affinities of the appendicular skeleton of *Oreopithecus bambolii* (IGF 11778). *J. Hum. Evol*. **16**, 445-456 (1987).

12. Harrison, T. New estimates of cranial capacity, body size and encephalization in *Oreopithecus bambolii*. *Am. J. Phys. Anthropol*. **78** (Suppl.), 237 (1989).

13. Conroy, G. C. Problems of body-weight estimation in fossil primates. *Int. J. Primatol*. **8**, 115-137 (1987).

14. Brunet M. *et al.* A new hominid from the upper Miocene of Chad, central Africa. *Nature* **418**, 145-51 (2002).

15. Zollikofer, C. P. E. *et al*. Virtual cranial reconstruction of *Sahelanthropus tchadensis*. *Nature* **434**, 755-759 (2005).

16. Uehara, S. & Nishida, T. Body weights of wild chimpanzees (*Pan troglodytes schweinfurthii*) of the Mahale Mountains National Park, Tanzania. *Am J. Phys. Anthropol*. **72**, 315-321 (1987).

17. Bienvenu, T. *et al*. The endocast of *Sahelanthropus tchadensis*, the earliest known hominid (7 Ma, Chad). *Am J. Phys. Anthropol*. **15,** 80-81 (2013).

18. Suwa, G. *et al*. The *Ardipithecus ramidus* skull and its implications for hominid origins. *Science*, **326**, 68e1-68e7 (2009).

19. Lovejoy, C.O. *et al*. The great divides: *Ardipithecus ramidus* reveals the postcrania of our last common ancestors with African apes. *Science*, **326**, 100-106 (2009).

20. Delson, E. *et al*. Body mass in Cercopithecidae (Primates, Mammalia): estimation and scaling in extinct and extant taxa. *Anthropol. Pap. Am. Mus*. **83**, 1-159 (2000).

21. Elton, S., Bishop, L .C. & Wood, B. Comparative context of Plio-Pleistocene hominin brain evolution. *J. Hum. Evol.* **41**, 1–27 (2001).

22. Krentz, H. in *Theropithecus: The Rise and Fall of a Primate Genus.* (ed. Jablonski, N. G.) 383-442 (Cambridge University Press, 1993).

23. Martin, R. D. in *Theropithecus: The Rise and Fall of a Primate Genus.* (ed. Jablonski, N. G.) 273-298 (Cambridge University Press, 1993).

24. Jolly, C. J. The classification and natural history of *Theropithecus* (*Simopithecus*) (Andrews, 1916), baboons of the African Plio-Pleistocene. *Bull. Br. Mus. Nat. Hist*. **22**, 1-123 (1972).
